# Estrogen Receptor-Alpha and p53 Status as Regulators of AMPK and mTOR in Luminal Breast Cancer

**DOI:** 10.3390/cancers13143612

**Published:** 2021-07-19

**Authors:** Nishant Gandhi, Chetan C. Oturkar, Gokul M. Das

**Affiliations:** Center for Genetics & Pharmacology, Department of Pharmacology and Therapeutics, Roswell Park Comprehensive Cancer Center, Buffalo, NY 14263, USA; ngandhi@carisls.com (N.G.); chetan.oturkar@roswellpark.org (C.C.O.)

**Keywords:** luminal breast cancer, estrogen receptor, p53, AMPK, mTOR, proximity ligation assay (PLA), endocrine therapy, therapeutic resistance, Fulvestrant, Everolimus

## Abstract

**Simple Summary:**

Endocrine therapy targeted against estrogen and the estrogen receptor is the main treatment modality for luminal breast cancer. Although a majority of patients respond to this treatment, one-third of all patients are resistant to this therapy. Hyperactive estrogen receptor along with alternative drivers such as mutations in the major tumor suppressor named p53 are known to contribute to resistance to therapy. The current study shines light on some of the mechanisms underlying the functioning of these players. A better understanding of these mechanisms will have important therapeutic implications by facilitating development of strategies to overcome therapeutic resistance by identifying novel targets and by combining drugs to effectively combat luminal breast cancer.

**Abstract:**

Luminal breast cancer (LBC) driven by dysregulated estrogen receptor-alpha (ERα) signaling accounts for 70% of the breast cancer cases diagnosed. Although endocrine therapy (ET) is effective against LBC, about one-third of these patients fail to respond to therapy owing to acquired or inherent resistance mechanisms. Aberrant signaling via ERα, oncogenes, growth factor receptors, and mutations in tumor suppressors such as p53 impinge on downstream regulators such as AMPK and mTOR. While both AMPK and mTOR have been reported to play important roles in determining sensitivity of LBC to ET, how the ERα-p53 crosstalk impinges on regulation of AMPK and mTOR, thereby influencing therapeutic efficacy remains unknown. Here, we have addressed this important issue using isogenic breast cancer cell lines, siRNA-mediated RNA knockdown, and different modes of drug treatments. Interaction of p53 with ERα and AMPK was determined by *in situ* proximity ligation assay (PLA), and endogenous gene transcripts were analyzed by quantitative real-time polymerase chain reaction (qRT-PCR). Further, the effect of concurrent and sequential administration of Fulvestrant–Everolimus combination on colony formation was determined. The studies showed that in cells expressing wild type p53, as well as in cells devoid of p53, ERα represses AMPK, whereas in cells harboring mutant p53, repression of AMPK is sustained even in the absence of ERα. AMPK is a major negative regulator of mTOR, and to our knowledge, this is the first study on the contribution of AMPK-dependent regulation of mTOR by ERα. Furthermore, the studies revealed that independent of the p53 mutation status, combination of Fulvestrant and Everolimus may be a viable first line therapeutic strategy for potentially delaying resistance of ERα+/HER2− LBC to ET.

## 1. Introduction

LBC accounts for ~70% of breast cancer cases diagnosed and is primarily driven by dysregulated ERα signaling [1,2]. The major therapeutic strategy being used at present is ET with ERα antagonists and agents that reduce the production of estrogen to inhibit LBC progression by interfering with ERα signaling. While ET has been responsible for reducing the relative rate of recurrence by 40%, treatment failure has been reported in ~30% of patients owing to acquired and inherent resistance mechanisms [3,4,5,6]. In particular, patients with tumors bearing mutations in p53 (mutation frequency: 12–30%) respond poorly to ET. Studies from our lab and others have suggested that the crosstalk between the ERα–p53 pathway may play an important role in mediating sensitivity to ET [7,8,9,10,11]. However, the mechanisms underlying the reduced response to ET observed in the mutant p53-expressing LBC tumors remain unclear.

The metabolic stress sensor AMPK, a target of ERα and p53, has been reported to repress several drivers of ET resistance such as HER2, EGFR, Akt, cyclin D1, CDK4, and ERα. In turn, these oncogenic drivers repress AMPK, suggesting that the repression of AMPK may play a critical role in mediating ET resistance [12,13,14,15,16,17,18,19,20]. While AMPK has been reported to repress ERα expression and transcriptional activity, AMPK’s regulation by ERα is not clear [21]. In breast cancer, two independent studies have reported that ERα may activate or repress AMPK in cell line models differing in their p53 mutational status [22,23]. While these studies indicated that p53 mutational status may play a role in ERα- mediated regulation of AMPK, conclusive evidence proving the same has not been reported. Similarly, wild type p53 has been shown to regulate AMPK by (1) increasing the expression of sestrins that subsequently bind to and activate AMPK and (2) by transcriptionally regulating AMPKβ levels (required for the formation of the AMPK trimeric complex). On the other hand, mutant p53 binds and represses AMPK phosphorylation [24,25,26,27,28]. Therefore, while both ERα and p53 have been shown to regulate AMPK independently, whether p53 and ERα influence each other’s ability to regulate AMPK is not known. Such crosstalk between p53 and ERα is suggested by earlier observations from our lab and others wherein ERα-p53 interactions were determined to affect target gene transcription and binding to partner proteins [9,29,30,31]. Furthermore, mTOR, a downstream target of ERα, p53, and AMPK, has been reported to play an important role in resistance of LBC to ET [32,33,34,35,36,37]. However, how the ERα-p53 crosstalk impinges on the regulation of AMPK and mTOR, thereby influencing therapeutic resistance remains unknown. Here, we have addressed this important issue using isogenic cell lines, siRNA-mediated RNA knockdown, and different modes of drug treatments. Finally, we have investigated how the dose frequency and sequencing of clinically approved ET (Fulvestrant) and mTOR inhibitors (Everolimus) impact treatment efficacy in LBC.

## 2. Materials and Methods

### 2.1. Cell Lines

Isogenic MCF7 cells that differ in p53 status were generated in our laboratory from parental MCF7 cells (expressing endogenous wtp53) (obtained from ATCC). MCF7-shp53 and MCF7-shGFP stable cell lines were previously generated in our laboratory by Dr. Wendy Swetzig [38] by infecting parental MCF7 cells with lentiviral particles expressing lentiviral plasmid shp53 pLKO.1 puro (plasmid # 19119, Addgene, Watertown, MA, USA) and lentiviral plasmid pLKO.1 puro GFP siRNA (plasmid # 12273, Addgene) (kind gift from Dr. Xinjiang Wang, Roswell Park Comprehensive Cancer Center), respectively. MCF-shp53 cells were transfected with pCR3.1-p53 R273H plasmid (constructed in our laboratory) to generate stable MCF7-mutp53 cell line. ZR-75-1 (expressing endogenous wtp53) and T-47D (expressing endogenous mutant-53) were from ATCC. MDA-MB-231-LM2.4Luc+ cells (referred to as MDA-MB-231 cells in the manuscript) were a kind gift from Drs. Robert Kerbel and John Ebos. All the cell lines were cultured in Dulbecco’s Modified Eagle’s medium (DMEM 10-013-CV, Corning, Tewksbury, MA, USA) supplemented with 10% fetal bovine serum (FBS 10437-028 Gibco, Thermo Fisher Scientific, Grand Island, NY, USA), penicillin (50 IU/mL), and streptomycin (50 g/mL) in an incubator maintained at 37 °C in humidified atmosphere containing 5% CO_2_. The cell lines were authenticated using short tandem repeat analysis at the Genomics shared facility at Roswell Park Comprehensive Cancer Center (Buffalo, NY, USA).

### 2.2. Reagents

Fulvestrant/ICI 182,780 and Everolimus/RAD001 were purchased from Tocris, Minneapolis, MN, USA. Fulvestrant and Everolimus were dissolved in 100% ethanol to a stock concentration of 10 mM and stored at −20 °C. Working stocks were prepared fresh at the time of treatment. Antibodies used are listed in Table S1.

### 2.3. Plasmid Expression/siRNA Transfections

Briefly, 300,000–500,000 cells were plated in a 60 mm dish. 24–48 h post seeding, 1000 ng of empty vector (pCR3.1)/ERα-N282G/wild type ERα plasmid was transiently transfected into cells using Lipofectamine 3000 (Thermo Fisher Scientific, Grand Island, NY, USA) as per the manufacturer’s protocol. Similarly, siRNA transfections were performed by transiently transfecting Silencer-Select Negative Control #1 siRNA or ERα-specific siRNA #12935-300 (Thermo Fisher Scientific, Grand Island, NY, USA) at a final concentration of 25 nM, into cells using Lipofectamine 3000 as per the manufacturer’s protocol. For experiments involving AMPK knockdown, control siRNA-A (sc-37007) or siRNA targeting AMPKα1/2 (sc44 45312) was transfected as described above to a final concentration of 50 nM. Cells were subsequently harvested 24–48 h post-transfection for further analysis.

### 2.4. Protein Lysis and Immunoblotting

Cells were washed with ice-cold 1× PBS and then harvested with the help of cell scrapers. The cells were pelleted by centrifugation at 2000 rpm for 5 min at 4 °C. The pellet was resuspended in 1× PBS, followed by another centrifugation. Subsequently, pellets were lysed with RIPA buffer with freshly added protease and phosphatase inhibitors (1× EDTA-free Complete Protease Inhibitor Cocktail, 10 mM sodium fluoride, and 1 mM sodium orthovanadate). Lysates were incubated on an end-to-end rotor at 4 °C for 1 h. The cell lysate was centrifuged for 10 min at 10,000 rpm at 4 °C to clear cell debris. The supernatant (containing the proteins) was transferred to a fresh tube and protein estimation was carried out using the Bradford method (Bio-Rad, Hercules, CA, USA) according to the manufacturer’s protocol. Protein lysates were boiled for 7 min after the addition of 4× protein sample buffer and run on an SDS-PAGE gel, followed by immunoblotting using ECL reagents (Thermo Fisher Scientific, Grand Island, NY, USA). The blots were exposed to X-ray films and developed using a Kodak X-OMAT processor.

### 2.5. Quantitative Real-Time PCR (qRT-PCR)

RNA from cells was isolated using Trizol (Thermo Fisher Scientific, Grand Island, NY, USA), according to the manufacturer’s protocol. Approximately 1 μg RNA was DNAse-treated (Amplification grade, Invitrogen) and used for cDNA synthesis using the iScriptTM cDNA synthesis kit (BioRad). Quantitative real-time PCR was performed using iTaqTM Universal SYBR Green Supermix (Bio-Rad, Hercules, CA, USA) in the ABI Prism 7300 Real time PCR machine (Applied Biosystems, Thermo Fisher Scientific, Grand Island, NY, USA). Data were analyzed using the ΔΔCt method. The transcripts of the genes of interest were first normalized to the transcripts of β-actin and then normalized to non-specific siRNA control treated MCF7 cells expressing wild type p53. PRKAA1 Forward Primer 5′-3′: GGA GCC TTG ATG TGG TAG GA, Reverse Primer 5′-3′: TTT CAT CCA GCC TTC CAT TC; ACTB-Forward Primer 5′-3′: ATG GGT CAG AAG GAT TCC TAT GT, Reverse Primer 5′-3′: AAG GTC TCA AAC ATG ATC TGG G.

### 2.6. Proximity Ligation Assay (PLA)

PLA was carried out using the Duolink II reagent kit ((#DUO92101 Millipore Sigma, St. Louis, MO, USA) as per the manufacturer’s protocol and as described previously [39]. The assay is a highly sensitive and specific in situ detection technique, that measures endogenous proteins, protein modifications and protein interactions. Mouse and rabbit primary antibodies specific to each pair of protein targets of interest were used.

### 2.7. Clonogenic Survival Assay

The clonogenic survival assay or the colony formation assay is an in vitro assay used to ascertain the ability of a single cell to grow into a colony (>50 cells). The assay is an in vitro visualization of a residual tumor cells’ (single cell) ability to form a tumor after treatment with chemical agents or ionizing radiation. One-thousand cells were seeded in a 60 mm cell culture dish. Cells were treated 24 h post-seeding or as depicted by the different dosing schemes. The concentration of Fulvestrant (0.92 nM) was based on the ED50 value determined for a 72 h growth inhibition assay of MCF7-shGFP cells. The efficacy of this concentration was further validated in the clonogenic assay. Although the ED50 value for Everolimus for a 72 h growth inhibition assay had also been determined (3.5 nM), a higher concentration of Everolimus (20 nM) based on its efficacy as a monotherapeutic and in combination with Fulvestrant for the purposes of the clonogenic assay was determined. Eleven days post-seeding, cells were fixed in 10% neutral buffered formalin for 30 min followed by staining with 0.5% crystal violet solution for 30 min (both at room temperature). Subsequently, plates were washed 4× in water to remove the excess stain and dried completely. Colonies were defined as containing at least 50 cells and were counted for analysis.

### 2.8. Statistical Analysis

GraphPad Prism 7.04 software was used to perform statistical analysis. Student’s *t*-test with Welch’s correction was used for comparing two groups. To determine statistical significance among the multiple experimental arms (multiple groups), the nonparametric version of the ANOVA, Kruskal–Wallis test followed by Dunn’s multiple comparisons test were performed. *p*-values less than 0.05 (*p* < 0.05) were deemed statistically significant and designated with *, *p* < 0.01 with **, *p* < 0.001 with ***, and *p* < 0.0001 with ****.

## 3. Results

### 3.1. Requirement of ERα for Repressing AMPK Phosphorylation Is Dependent on p53 Mutational Status

**Figure 1:**
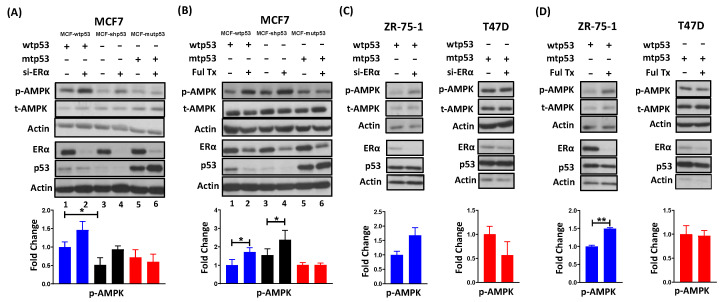
Requirement for repression of AMPK phosphorylation by ERα in LBC is dependent on p53 status (wild type p53 versus mutant p53). MCF7 isogenic cells differing in their p53 status (MCF7-wtp53, MCF7-shp53, and MCF7-mutp53) (**A**,**B**), ZR-75-1, and T47D (**C**,**D**) were treated with (**A**,**C**) siRNA (25 nM) targeting ERα or (**B**,**D**) Fulvestrant (100 nM) for 48 h. Subsequently, cells were lysed, and immunoblotting analysis was performed to probe for AMPK phosphorylation (p-AMPK), ERα, p53, and actin. Panels below each immunoblot represent graphical representation of densitometric analysis of p-AMPK levels normalized to actin and then to non-specific silencing siRNA/Vehicle treatment. Values reflect mean ± standard deviation of at least three biological replicates. * *p*-value < 0.05, demonstrate statistical significance by One-way ANOVA (**A**,**B**). ** *p*-value < 0.01, demonstrate statistical significance by Students *t*-test (**C**,**D**). Quantitation of protein expression levels of total AMPK (t-AMPK) (Figure S1-Part A), ERα (Figure S1-Part B), and p53 (Figure S1-Part C) are in the Supplementary section. wtp53: wild type p53; mutp53: mutant p53; Ful: Fulvestrant.

AMPK is activated by phosphorylation on its Thr172 residue and both ERα and p53 have been independently reported to regulate AMPK. In order to determine whether these proteins influence each other’s ability to regulate AMPK phosphorylation (p-AMPK), we used MCF7 isogenic cells differing in their p53 mutational status (MCF7-wtp53, MCF7-shp53, and MCF7-mutp53) and modulated ERα expression in these cells by transiently transfecting ERα-specific siRNA or by treatment with Fulvestrant, a selective estrogen receptor degrader (SERD). Subsequently, p-AMPK expression was assayed via immunoblotting. We determined that a reduction in ERα levels resulted in an induction of p-AMPK in MCF7 cells expressing wild type p53 but not mutant p53 (Figure 1A,B, compare lanes 1 vs. 2 and 5 vs. 6). These results were further validated in additional LBC cell lines expressing endogenous wild type p53 (ZR-75-1 and endogenous mutant p53 (T47D). Here, too we found that a reduction in ERα levels induced p-AMPK in ZR-75-1 cells but not in T47D cells (Figure 1C,D). Importantly, the induction of p-AMPK upon the loss of ERα in MCF7 cells lacking wild type p53 suggested that ERα may repress AMPK in a p53-independent manner as well (Figure 1A,B, compare lanes 3 vs. 4). [see quantitation of protein expression levels of total AMPK (t-AMPK) (Figure S1-Part A), ERα (Figure S1-Part B), and p53 (Figure S1-Part C)]. Thus, the reason for ERα being unable to decrease p-AMPK levels in mutant p53-expressing cells is because mutant p53 by itself may be an efficient repressor of AMPK phosphorylation. In other words, in the mutant p53 expressing cells, loss of ERα did not induce p-AMPK levels as mutant p53 sustains the repression of AMPK phosphorylation. Therefore, while ERα may be required to repress AMPK phosphorylation in wild type p53 expressing LBC, it is not required in LBC-expressing mutant p53 (Figure 1).

### 3.2. ERα Impairs Mutant p53’s Ability to Bind AMPK

**Figure 2:**
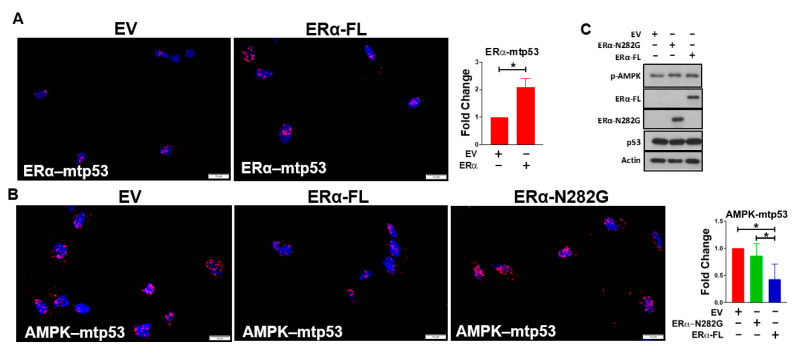
ERα impairs mutant p53’s ability to bind to AMPK. MDA-MB-231 cells were transfected with ERα-FL (full length), ERα-N282G (truncated at aa282, thus lacking the domain required for p53 interaction) or empty vector to assess the impact of ERα on mutant p53-AMPK interactions using the PLA. PLA (**A**) for Erα–mutant p53 interaction and (**B**) for AMPK–mutant p53 interaction. Further, immunoblotting analysis was performed to determine whether the expression of ERα altered AMPK phosphorylation in the MDA-MB-231 cells (**C**). PLA interactions were assayed for the following interacting pairs: total-AMPK-mtp53 and ERα-mtp53. The interactions were quantified and the graphed values reflect mean ± standard deviation of at least 3 biological replicates. * *p*-value < 0.05 demonstrates statistical significance by Student’s *t*-test (**A**) or by One-way ANOVA (**B**). Quantitation of protein levels of p-AMPK, ERα, and p53 (Figure 2C) are shown in Figure S2C. PLA: proximity ligation assay; aa: amino acid; EV: Empty vector.

Mutant p53 has been reported to bind to and repress AMPK phosphorylation [28]. As previous studies from our lab have demonstrated that ERα binds to and antagonizes wild type p53 [9,29,30,40], we hypothesized that ERα will bind mutant p53 and this interaction will attenuate mutant p53’s ability to bind to AMPK. In order to test this hypothesis, we used MDA-MB-231 cells, a triple-negative breast cancer cell line (TNBC) model, as these cells lack ERα and express mutant p53 (R280K). The cells were transfected with domains of ERα that differ in their ability to bind p53. This allowed us to test whether ERα-mutant p53 interaction influenced the ability of mutant p53 to bind AMPK. The PLA which allows the visualization of protein interactions in situ, showed that the ectopic expression of ERα in MDA-MB-231 cells resulted in an increase in ERα-mutant p53 interactions (Figure 2A) and a decrease in mutant p53–AMPK interactions (Figure 2B). These results suggested that by binding mutant p53, ERα attenuated mutant p53’s ability to bind AMPK. The interactions between mutant p53 and AMPK were observed in both the cytoplasmic and the nuclear compartments. Although both proteins can localize in the cytosol and the nucleus, given the high levels of nuclear localization of mutant p53, a majority of the mutant p53–AMPK interactions appeared to be in the nuclear region. A similar spatial distribution of ERα–mutant p53 interaction was observed.

Prior studies from our lab identified that the c-terminal domain (CTD) of ERα (aa283–595) is required for binding p53 [29]. The deletion mutant N282G ERα protein in which the CTD of ERα (aa283–595) is absent, consequently lacks the domain required to bind p53. In order to test whether ERα–mutant p53 interaction is required for attenuating mutant p53’s ability to bind AMPK, we transfected the ERα-N282G mutant in MDA-MB-231 cells and assayed its impact on mutant p53–AMPK interaction using the PLA. In accordance with our hypothesis, the mutant p53–AMPK interactions in MDA-MB-231 cells transfected with ERα-N282G mutant were similar to those observed in the empty vector control transfected cells (Figure 2B). Furthermore, the mutant p53–AMPK interactions in MDA-MB-231 cells transfected with ERα-N282G mutant and the empty vector control were significantly greater than those observed in ERα wild type/full length transfected cells. These results suggested that ERα interaction with mutant p53 is required for the attenuation of mutant p53’s ability to bind to AMPK.

Further, as the interaction of mutant p53 with AMPK is required for the repression of AMPK phosphorylation, we investigated whether the ERα-mediated attenuation of mutant p53–AMPK interaction resulted in a de-repression of AMPK phosphorylation. We observed that ectopic expression of ERα in MDA-MB-231 cells did not result in de-repression of AMPK phosphorylation (Figure 2C), suggesting that ERα may itself (independent of p53) repress AMPK phosphorylation, thereby countering the effect of ERα on inactivating mutant p53. These results complimented the results observed in Figure 1A,B which demonstrated that in the absence of p53, ERα likely repressed AMPK phosphorylation. Therefore, in the MDA-MB-231 model, while overexpressing ERα led to a decrease in mutant p53-AMPK interaction, the consequent lack in induction of p-AMPK was potentially due to the increase in ERα mediated repression of AMPK. Quantitation of protein levels of p-AMPK, ERα, and p53 (Figure 2C) are shown in Figure S2C.

### 3.3. ERα Regulates mTOR Signaling through AMPK-Dependent and -Independent Mechanisms

**Figure 3:**
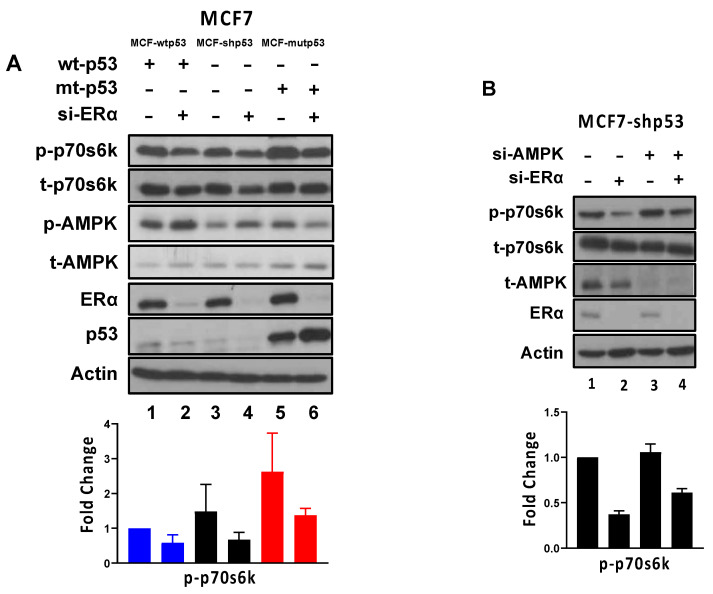
ERα and p53 regulate mTOR pathway. MCF7 isogenic cells (MCF-wtp53, MCF7-shp53, and MCF7-mutp53) differing in their p53 mutational status were treated with (**A**) siRNA targeting ERα (25 nM) for 48 h. (**B**) MCF7-shp53 cells were treated with siRNA targeting ERα (25 nM) or AMPKα1/2 (50 nM) or both for 48 h. Subsequently, cells were lysed, and immunoblotting analysis was performed probing for p-p70s6k (a downstream target of mTOR activation), total p70s6k (t-p70s6k), p-AMPK, total AMPK (t-AMPK), ERα, and p53. Representative blots for actin are shown. Quantitation of t-p70s6k, ERα, and AMPK protein expression levels are shown in Figure S3A,B. Panel below immunoblot (**A**) represents graphed densitometric analysis of p-p70s6k levels normalized to actin and then to non-specific silencing siRNA treatment of the wild type p53 expressing condition. Panel below immunoblot (**B**) represents graphed densitometric analysis of p-p70s6k levels normalized to actin and then to non-specific silencing siRNA treatment. Values reflect mean ± standard deviation of two-three biological replicates.

Prior reports have detailed how ERα and p53 separately regulate mTOR signaling [24,27,28,41,42,43,44,45]. In order to investigate whether ERα and p53 coordinately regulate mTOR signaling, we used the MCF7 isogenic cell line model system to assess whether p53 mutational status influenced ERα’s ability to regulate mTOR. ERα expression was regulated by transfecting the cells with ERα-specific siRNA and mTOR signaling activity was assessed by immunoblot analysis of phosphorylated p70s6k (p-p70s6k) protein (an important downstream target of activated mTOR, and therefore, a surrogate of mTOR activity). Consistent with previous reports, we observed enhanced mTOR signaling in mutant p53-expressing cells compared to wild type p53-expressing cells, despite comparable levels of p-AMPK. This was evidenced by enhanced p-p70s6k levels in MCF7-mutp53 cells compared to MCF7-wtp53 and MCF7-shp53 cells (Figure 3A, compare lane 5 with lanes 1 and 3). Further, in cells transfected with ERα-specific siRNA, p-p70s6k levels were lower (lanes 2, 4, and 6) compared to the levels in cells transfected with non-specific siRNA control (lanes 1, 3, and 5) independent of p53 mutational status. These results suggested that ERα by itself is a potent activator of mTOR signaling in these cells. It is noteworthy that both the basal levels and post-ERα siRNA treatment levels of p-p70s6k were higher in the MCF7-mutp53 cells compared to MCF7-wtp53 cells and MCF7-shp53 cells (Figure 3A) suggesting heightened basal mTOR activity in cells expressing mutant p53 (Figure 3A, lanes 5 and 6). Quantitation of t-p70s6k, ERα, and AMPK protein expression levels are shown in Figure S3A,B.

As AMPK is a major negative regulator of mTOR, we investigated the contribution of the ERα-mediated repression of AMPK on its ability to regulate mTOR activity. In order to assess mTOR regulation by ERα via AMPK-dependent mechanisms, we transfected MCF7-shp53 cells with siRNA targeting ERα and AMPK both alone and in combination. We observed that knocking down ERα by itself or in combination with AMPK led to a reduction in p-p70s6k levels (Figure 3B). However, the p-p70s6k levels when both ERα and AMPK were knocked down were greater than when only ERα was knocked down, indicating that AMPK played an important role in mediating ERα’s regulation of mTOR. Of note, knocking down AMPK by itself did not considerably increase p70s6k levels. We calculated the contribution of the AMPK dependent regulation of mTOR by ERα to be ~30% of the total regulation of mTOR by ERα.

### 3.4. Effect of Combined mTOR Inhibition and ET on Clonogenic Survival: Drug Dosing Scheme

**Figure 4:**
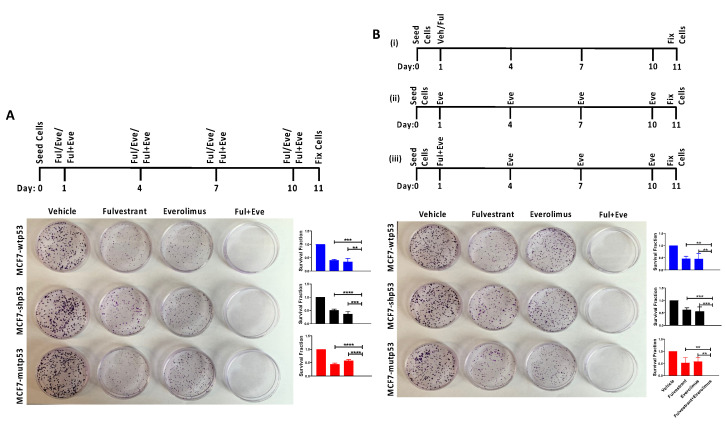
Effect of different modes of combining Fulvestrant and Everolimus on the clonogenic survival of MCF7 isogenic cell lines. (**A**) Mono- or combination-therapy with multiple doses of Fulvestrant and Everolimus. The schematic of the treatment is shown on the top. MCF7 isogenic cells differing in their p53 mutational status (MCF-wtp53, MCF7-shp53, and MCF7-mutp53) were seeded at low density on day 0. Cells were then treated with Fulvestrant (0.92 nM) and Everolimus (20 nM) as mono- or combined therapy 24 h post-seeding (day 1) and subsequently both drugs were replenished every 72 h (days 4, 7, and 10). Cells were then fixed 11 days post-seeding, and colonies were counted for further analysis. The effect of the drug treatment on surviving colonies is pictorially and graphically represented. Values reflect mean ± standard deviation of three biological replicates. ** *p*-value < 0.01 *** *p*-value < 0.001 and **** *p*-value < 0.0001 demonstrate statistical significance by One-way ANOVA. Ful: Fulvestrant, Eve: Everolimus. (**B**) Determining the effect of multiple-dose treatment of Everolimus added to a single dose of Fulvestrant. The treatment schematic is shown on the top panel. MCF7 isogenic cells differing in their p53 mutational status, were seeded at low density on day 0. Cells were then treated with Fulvestrant (0.92 nM) and Everolimus (20 nM) as monotherapy or combination therapy 24 h post seeding on day 1 (i, ii, and iii, respectively). Given the short half-life of the drug, Everolimus was added every 72 h (days 4, 7, and 10) to the Everolimus monotherapy arm (i) and to the combination therapy arm (iii), while Fulvestrant was added only once (day 1) in Fulvestrant monotherapy and in the combination therapy arms, as shown in the schematic (i, iii). Cells were then fixed 11 days post-seeding, and colonies were counted for further analysis. The effect of the drug treatment on surviving colonies is pictorially and graphically represented. This dosing scheme closely mimics the treatment of the two drugs in the clinic, where Fulvestrant is given once every 28 days and Everolimus is given daily. Values reflect mean ± standard deviation of three biological replicates. ** *p*-value < 0.01, *** *p*-value < 0.001 demonstrate statistical significance by one-way ANOVA. Ful: Fulvestrant, Eve: Everolimus.

The mTOR pathway hyperactivation has been reported to play an important role in attenuating sensitivity of LBC tumors to ET. Consequently, mTOR inhibitors were reported to enhance the efficacy of ET [34,35,36,46,47,48]. Currently, the mTOR inhibitor Everolimus is used in combination with ET with significant benefit over ET alone in ERα+/Her2− advanced breast cancer patients who have failed prior ET [46,47,48]. However, the efficacy of the drug combination in the clinic is considerably hindered by the increased treatment related adverse effects (TRAE) caused as a result of adding Everolimus to ET [46,47,49,50,51,52]. Furthermore, the benefits of combining ET and mTOR inhibitors in ET naïve/sensitive patients is still being evaluated [49,53]. With the long-term goal of reducing TRAE and improving the efficacy of the drug combination, we provided an in vitro platform to test the impact of dosing scheme on clonogenic survival in our MCF7 isogenic cell lines. After seeding these cells at low density, we treated the cells with Fulvestrant and Everolimus as monotherapy and combined therapy 24 h post-seeding. Subsequently, the drugs were replenished every 72 h until the end of the experiment as depicted in the treatment schematic (Figure 4A). Upon analyzing the surviving colonies at the end of the experiment, we observed that both Fulvestrant and Everolimus monotherapy significantly reduced the clonogenic survival of MCF7 cells. Furthermore, combined Everolimus and Fulvestrant therapy reduced clonogenic survival of MCF7 cells compared to either monotherapy, independent of p53 mutational status. Given the long half-life of Fulvestrant, we investigated whether adding only Everolimus to the combination treatment every 72 h, would demonstrate similar efficacy to replenishing both drugs every 72 h (Figure 4B). In accordance with our hypothesis, we observed that adding Everolimus every 72 h to Fulvestrant (one dose) in the combination treatment reduced the clonogenic survival of MCF7 cells to a similar extent as compared to the multiple dosing of both drugs (compare Figure 4A,B).

### 3.5. Effect of Combined mTOR Inhibition and ET on Clonogenic Survival: Drug Sequencing

**Figure 5:**
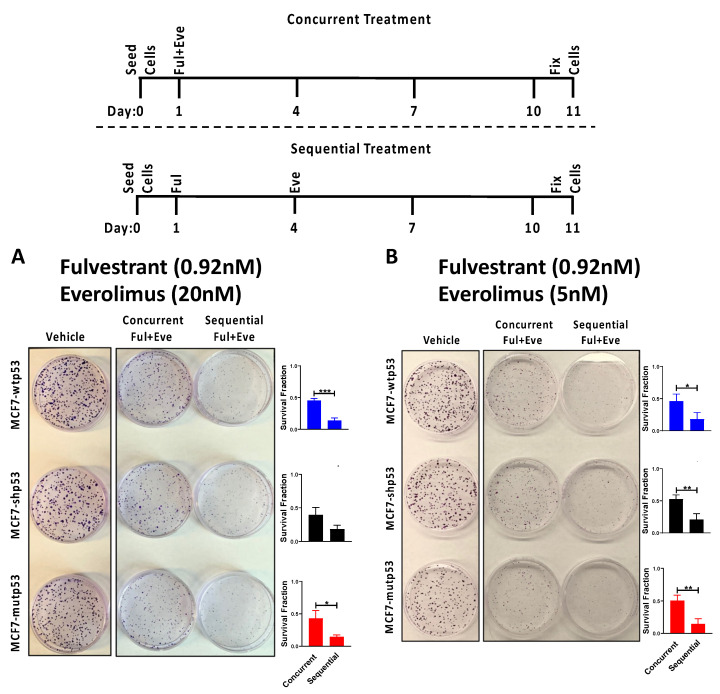
Sequential treatment of Fulvestrant and Everolimus is more efficacious than concurrent treatment in inhibiting clonogenic survival of LBC cells. Concurrent vs. sequential treatment of (**A**) Fulvestrant (0.92 nM) and Everolimus (20 nM) and (**B**) Fulvestrant (0.92 nM) and Everolimus (5 nM). Schematic of the dosing scheme is shown on the top. MCF7 isogenic cells differing in their p53 mutational status (MCF-wtp53, MCF7-shp53 and MCF7-mutp53), were seeded at low density on day 0. Cells were then treated with Fulvestrant (0.92 nM) and Everolimus as combination therapy 24 h post-seeding. For the combination treatment in the concurrent dosing scheme, cells were treated with both drugs at the same time on day 1. In the sequential dosing scheme Fulvestrant was treated 24 h post-seeding (day 1) and subsequently Everolimus was added 72 h (day 4) post Fulvestrant treatment in the combination treatment. Cells were then fixed 11 days post-seeding, and colonies were counted for further analysis. The effect of the dosing scheme on surviving colonies is pictorially and graphically represented. Values reflect mean ± standard deviation of three biological replicates. * *p*-value < 0.05, ** *p*-value < 0.01, *** *p*-value < 0.001 demonstrate statistical significance by Students *t*-test. Ful: Fulvestrant, Eve: Everolimus.

Previous studies have reported that mTOR inhibition induces Akt phosphorylation which is enhanced in the presence of E2-ERα signaling [54,55,56]. Additionally, ERα promotes PI3K/Akt activation through genomic and non-genomic mechanisms. By degrading ERα, Fulvestrant treatment attenuates both ERα-induced and mTOR inhibition-induced Akt phosphorylation. Therefore, we investigated whether sequencing Fulvestrant and Everolimus as depicted in Figure 5A, would enhance the efficacy of the combination therapy. When we sequenced the drugs such that Fulvestrant was administered first (24 h post seeding) and a single dose of Everolimus was added 72 h post-Fulvestrant treatment, we observed that the combination therapy was more efficacious than when the cells were treated concurrently (Figure 5A). These results indicated that when considering the combination therapy for patients, Everolimus administered as a single dose but sequenced (post-Fulvestrant treatment) could be superior to single dose concurrent treatment (with Fulvestrant).

As sequencing Everolimus post-Fulvestrant treatment significantly reduced clonogenic survival compared to concurrent administration of the two drugs, we investigated whether drug sequencing facilitated a dose de-escalation of Everolimus while maintaining comparable therapeutic efficacy. Interestingly, MCF7 cells treated with Fulvestrant followed by Everolimus at a 4-fold lower dose (5 nM) reduced the clonogenic survival (Figure 5B) to a similar extent to that observed with MCF7 cells treated with Fulvestrant followed by Everolimus at 20 nM. Here, too, the sequential treatment displayed enhanced efficacy as compared to the concurrent treatment. These results suggested that drug sequencing may facilitate dose de-escalation.

## 4. Discussion

As one of the most frequently mutated genes in breast cancer, p53 has been reported to be of both prognostic and predictive significance in LBC [57]. In particular, LBC patients with tumors bearing mutations in p53 respond poorly to ET [7,8,9,10,11]. In order to better define the mechanisms underlying the reduced response to ET in these tumors, we investigated how ERα (the primary target of ET) and p53 coordinately regulate AMPK and mTOR pathway activity—two important determinants of efficacious ET. In breast cancer, ERα mediated regulation of AMPK has been inconclusive [22,23]. As the discrepant regulation of AMPK by ERα may be borne out of cell line models differing in their p53 status (among other genetic variations), we investigated the role of p53 mutational status on ERα mediated regulation of AMPK. We have demonstrated that ERα represses AMPK phosphorylation in LBC cells in a p53-independent manner. However, in mutant p53 expressing cells, mutant p53 represses AMPK in the absence (and presence) of ERα. Therefore, ERα is required to repress AMPK in LBC expressing wild type but not mutant p53. We further identified that ERα may bind mutant p53, thereby attenuating mutant p53–AMPK interactions. The attenuation of mutant p53–AMPK interaction by ERα did not result in an induction of AMPK activation as expected based on reports by Zhou et al., substantiating our hypothesis that ERα may independently repress AMPK. There are several mechanisms by which ERα may repress AMPK. The liver kinase B1 is one of the major kinases responsible for regulating AMPK activity. As demonstrated by Zhou et al., it is possible that similar to mutant p53 ER may bind to AMPK and prevent LKB1-based phosphorylation [28]. Similarly, as demonstrated by Mauro et al., ERα may also bind and sequester LKB1, thereby repressing AMPK phosphorylation [23]. Finally, through its functional antagonism of p53, ERα may indirectly repress LKB1 transcription and consequently repress AMPK phosphorylation [58,59]. Interestingly, our studies demonstrated that while ERα may repress AMPK transcription in both wild type and mutant p53 expressing MCF7 cells, a similar repression of AMPK was not observed in MCF7 cells lacking intact wild-type p53 (Figure 2A), suggesting that p53 (either wild type or mutant) may be required for ERα mediated regulation of AMPK transcription. We recognize that not all p53 mutants are equal and hence the “net” impact of ERα and p53 on AMPK regulation may likely be determined by the specific type of p53 mutation. From a therapeutic standpoint, identifying the activation status of AMPK may serve as an important determinant of the type of adjuvant therapy to be used with ET in LBC. Our observation that Fulvestrant treatment led to an induction of AMPK in wild type but not in mutant p53-expressing LBC suggests that inhibitors of pathways activated by AMPK such as fatty acid oxidation inhibitors may be more efficacious in the prior cell type as compared to the later.

Activated AMPK is a negative regulator of mTOR pathway activity. While prior studies have reported multiple points of interaction between ERα and mTOR pathways, to our knowledge, this study is the first to report the contribution of AMPK-dependent regulation of mTOR by ERα [43,44,60,61,62]. Using MCF7-shp53 cells (to remove any confounding effects of p53 mediated regulation of AMPK), we determined that ERα may regulate mTOR pathway activity in an AMPK-dependent manner. We estimated the contribution of this pathway to account for ~30% of the total regulation of mTOR by ERα, underscoring the significance of this mechanism of regulation. Intriguingly, mutant p53 expression was upregulated in cells where ERα was knocked down (Figure 4A). Further studies are needed to understand the underlying mechanisms and functional relevance of this effect. As mentioned above, the hyperactivation of the mTOR pathway has been reported in LBC that respond poorly to ET. Consequently, mTOR inhibitors have been clinically approved for use in combination with ET for treating LBC that have progressed on prior ET [46,47,48]. While the addition of mTOR inhibitors to ET has significant therapeutic benefits as compared to ET alone, substantial increase in TRAE have been reported as a consequence. The increase in TRAE due to the addition of mTOR inhibitors to ET has resulted in several instances of dose reductions and abrupt treatment discontinuations [46,47,49,50,51,52]. As a step towards developing strategies to tackle this problem, we tested the impact of dose frequency and sequencing of Everolimus and Fulvestrant on clonogenic survival in MCF7 isogenic cells. We observed that the combined efficacy of Fulvestrant and Everolimus when both drugs were replenished every 72 h was similar in efficacy to when only Everolimus was added to the combination every 72 h. These observations are in line with the expected half-lives of the drugs and the dosing scheme depicted in Figure 5A closely mimics the current dosing in the clinical setting. We further demonstrated that the drug sequencing, i.e., either concurrent or sequential administration, may significantly impact treatment efficacy. Sequential administration of a single dose of Everolimus 72 h post-Fulvestrant treatment resulted in an ~80% reduction in clonogenic survival as compared to ~40–50% reduction when both drugs were administered concurrently. This stressed the importance of drug sequencing when designing a treatment regimen. Importantly, while the sequential treatment reduced the clonogenic survival by 80%, the multiple dose Everolimus treatment (with one dose of Fulvestrant) had a ~100% reduction in clonogenic survival as compared to the vehicle treatments (Figure 4). In the multiple dose scheme of Everolimus, 4 doses of Everolimus (Figure 4B) were added to Fulvestrant in the combination arm as compared to 1 dose of Everolimus (Figure 5A or 5B) being added to Fulvestrant in the combination arm of the sequential dosing scheme. These results provide a platform to perform further dose frequency and dose sequence-optimization studies in an in vivo model of LBC to ascertain the impact on tumor inhibition. Reducing the dosing frequency of Everolimus while maintaining comparable tumor growth inhibition would likely translate to clinically reduced TRAE, extended treatment duration and improved therapeutic efficacy.

## 5. Conclusions

In LBC, the requirement of ERα to repress AMPK is dependent on the mutational status of p53. In the wild type p53 context, ERα is required for the repression of AMPK, whereas AMPK repression in the context of mutant p53 does not require ERα. While ERα can activate mTOR independent of AMPK levels and p53 mutational status, activation of mTOR by mutant p53 is augmented by ERα. This is the first study to report the contribution of AMPK-dependent regulation of mTOR by ERα, and based on our findings, ERα-p53 crosstalk appears to impinge on mTOR pathway. Sequential treatment with Fulvestrant and Everolimus is more effective than concurrent treatment with these agents in suppressing LBC cell proliferation, a finding that has important clinical implications in delaying therapeutic resistance in luminal breast cancer.

## Data Availability

The data presented in this study are openly available.

## Supplementary Materials

The following are available online at https://www.mdpi.com/article/10.3390/cancers13143612/s1, Figure S1-Part A, Figure S1-PartB, Figure S1-PartC, Figure S2C, Figure S3 A,B, Table S1: List of Antibodies.
