# Supplementary material for: Estrogen Receptor-Alpha and p53 Status as Regulators of AMPK and mTOR in Luminal Breast Cancer
Source: Cancers (Basel). 2021 Jul 19;13(14):3612. doi: 10.3390/cancers13143612 (PMC8306694; doi:10.3390/cancers13143612)
Supplement: Supplementary file 1 [file cancers-13-03612-s001.zip › cancers-1287357-SI.pptx]

## Slide 1
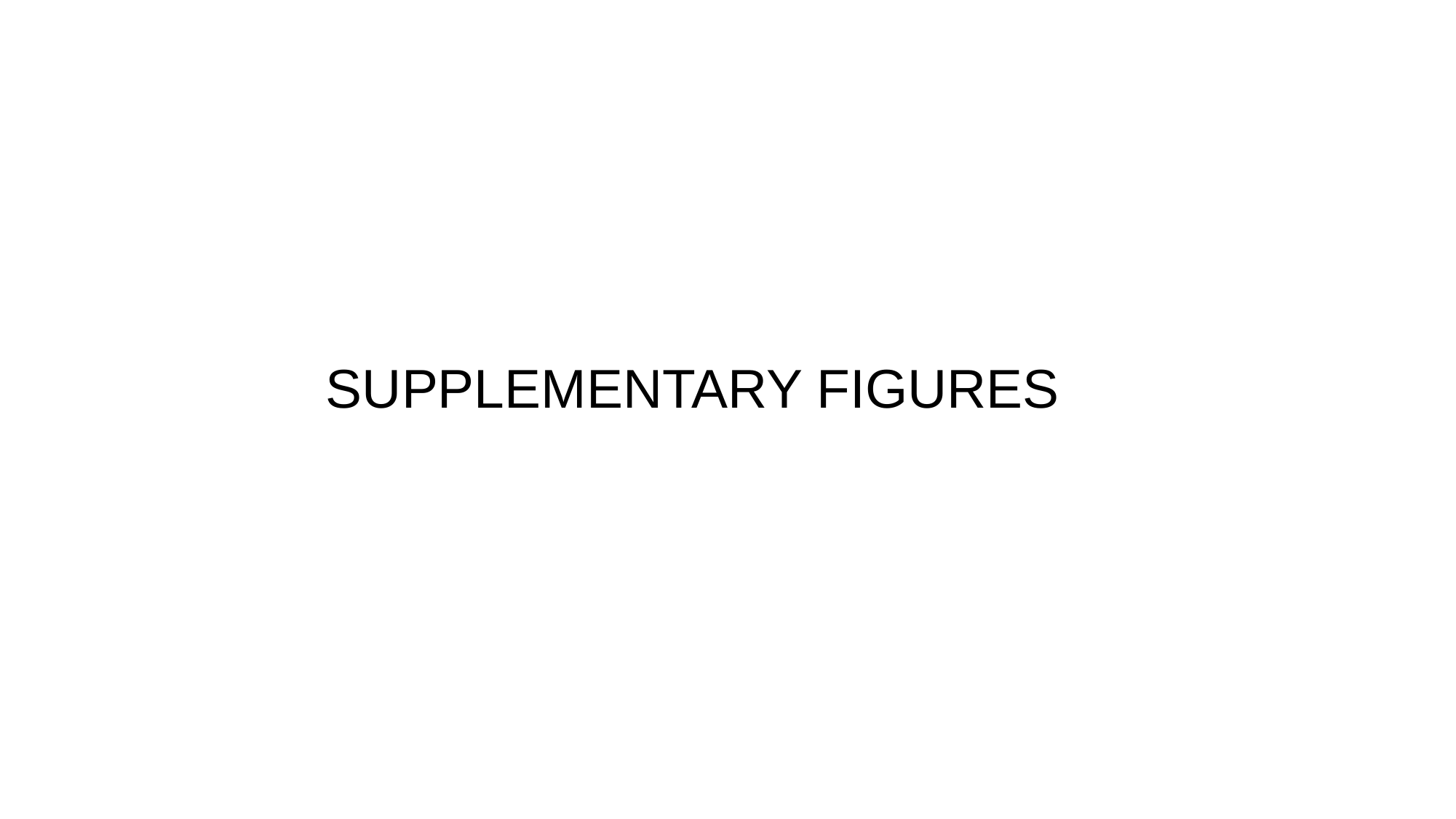

SUPPLEMENTARY FIGURES

## Slide 2
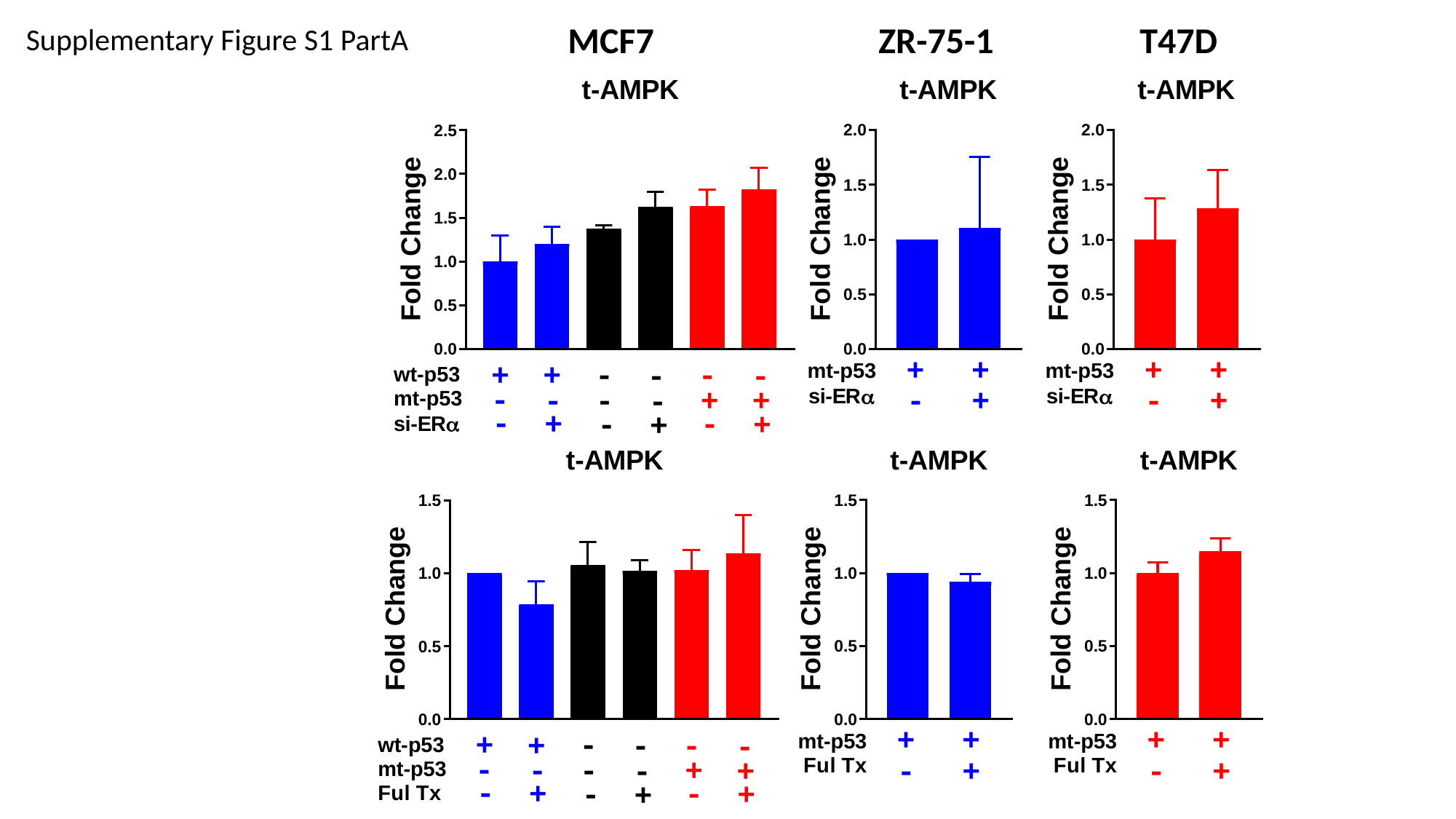

MCF7
T47D
ZR-75-1
Supplementary Figure S1 PartA

## Slide 3
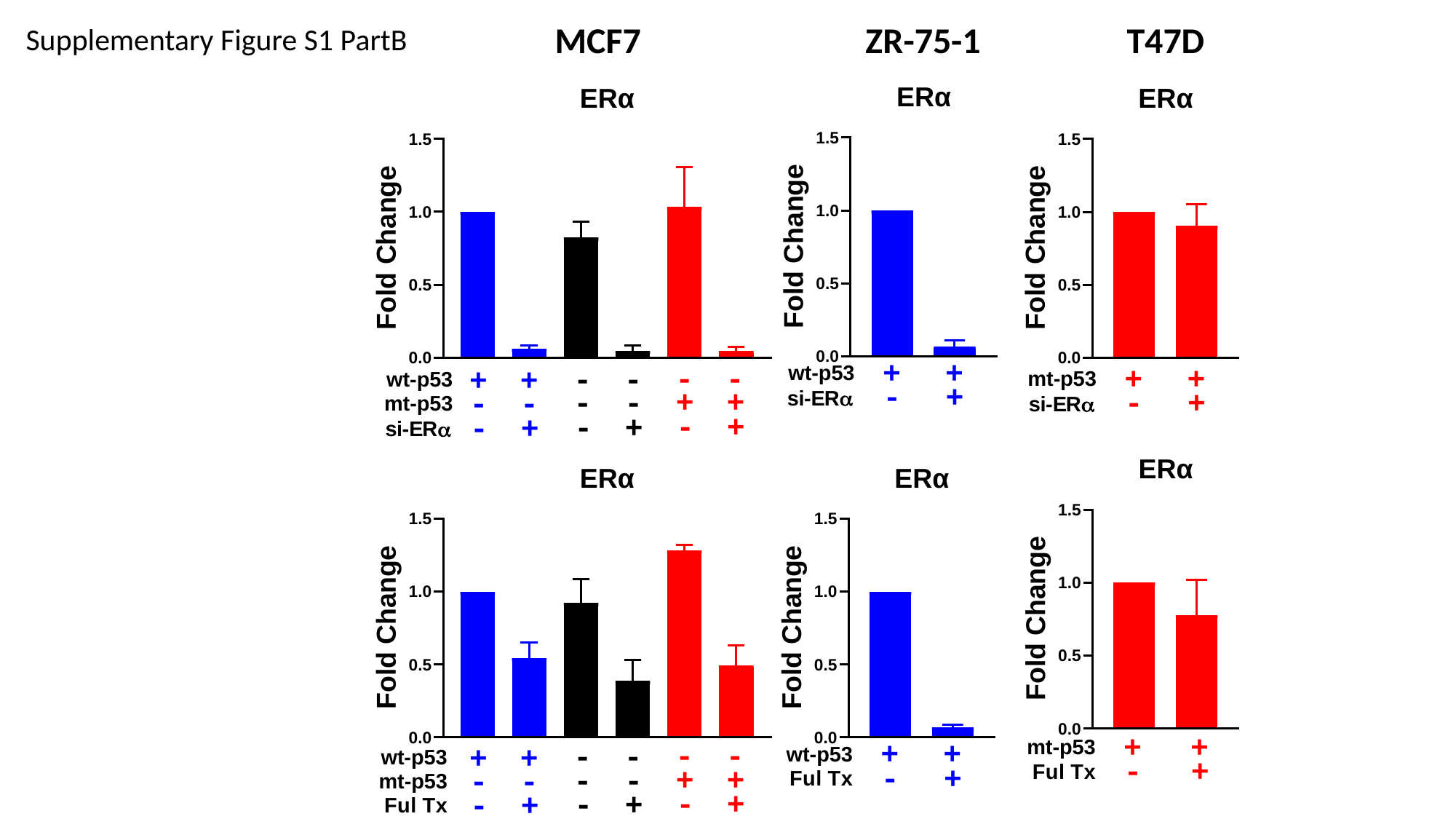

MCF7
T47D
ZR-75-1
Supplementary Figure S1 PartB

## Slide 4
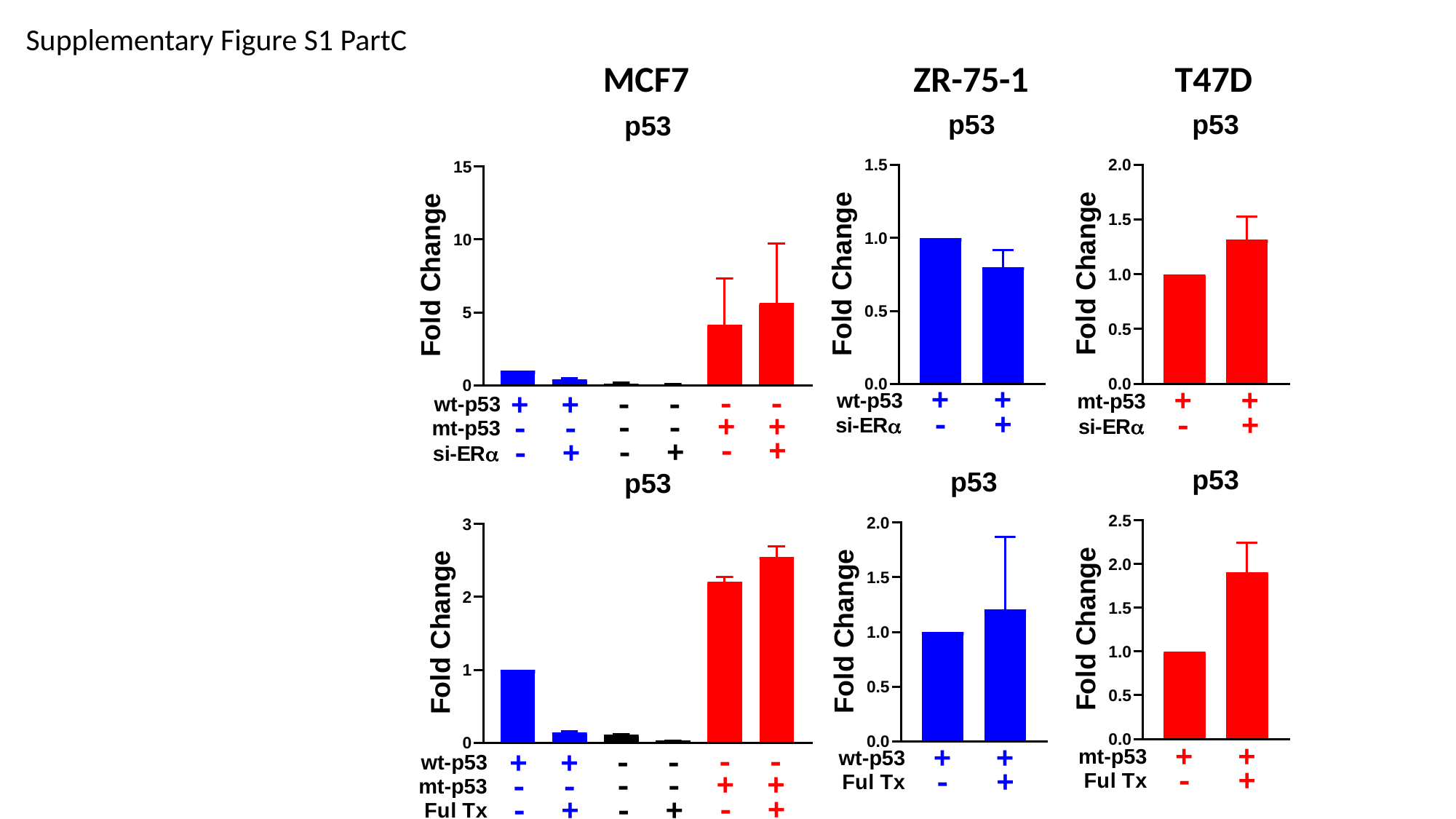

Supplementary Figure S1 PartC
MCF7
T47D
ZR-75-1

## Slide 5
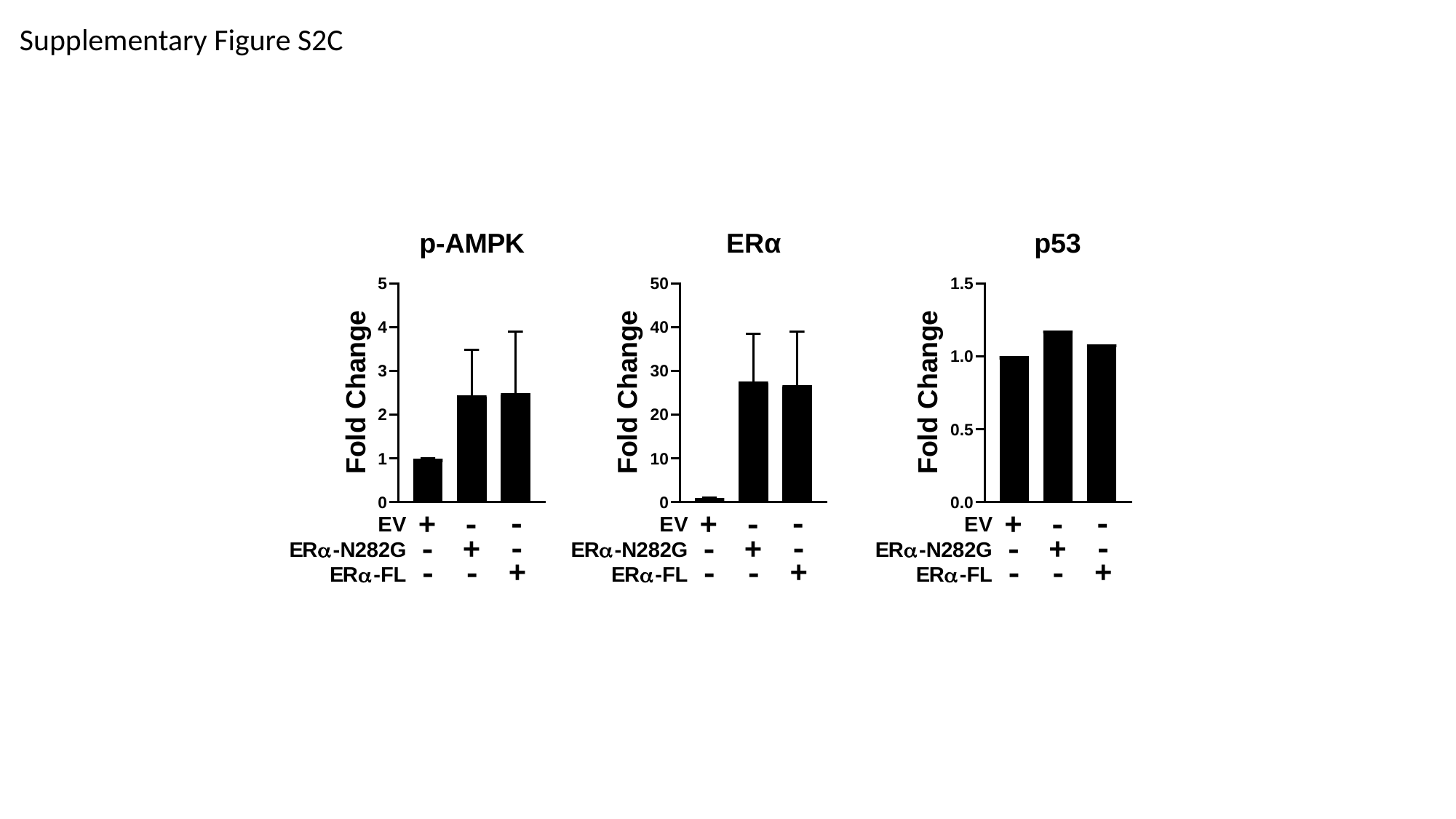

Supplementary Figure S2C

## Slide 6
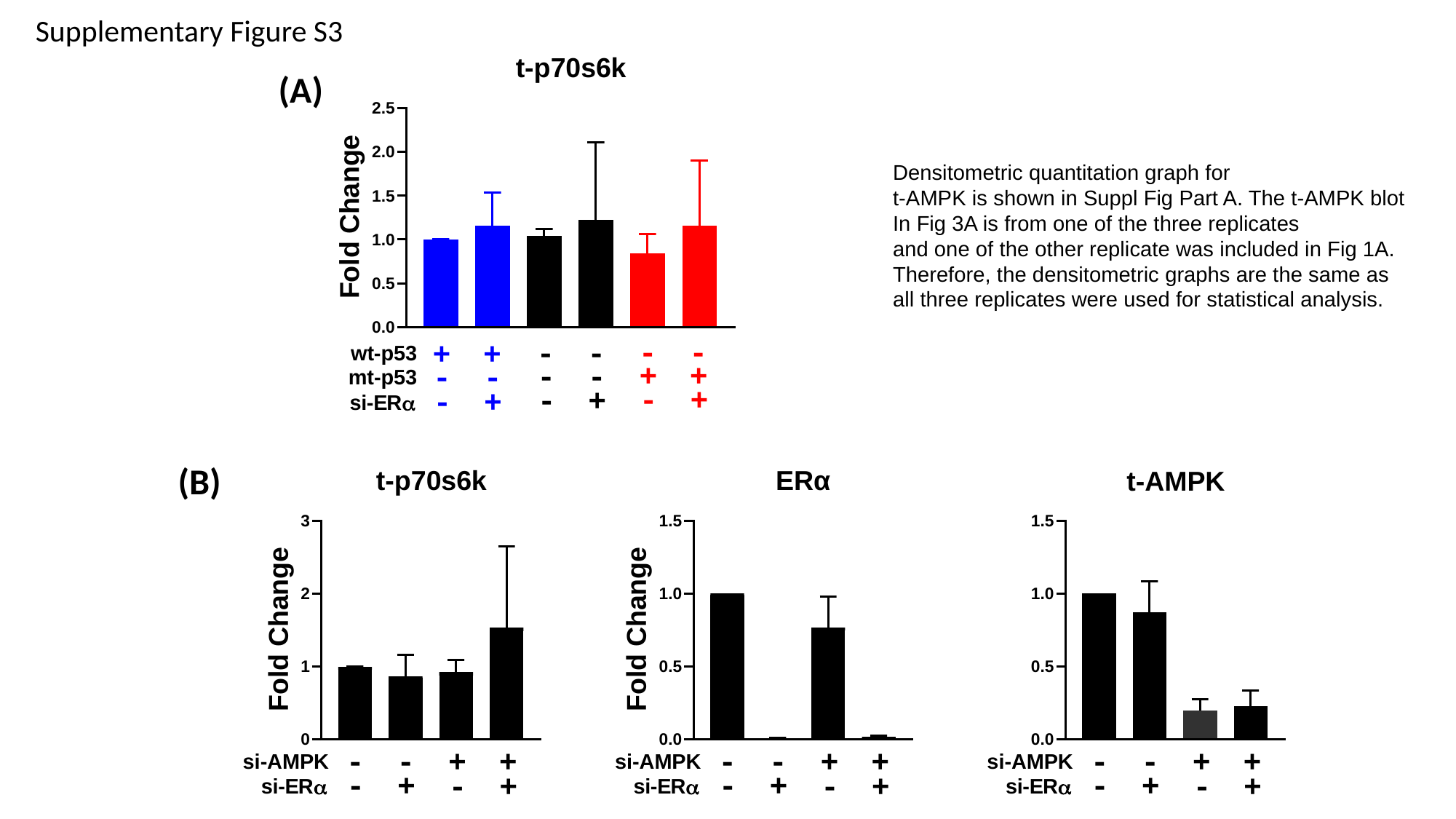

Supplementary Figure S3
(A)
Densitometric quantitation graph for
t-AMPK is shown in Suppl Fig Part A. The t-AMPK blot
In Fig 3A is from one of the three replicates
and one of the other replicate was included in Fig 1A.
Therefore, the densitometric graphs are the same as
all three replicates were used for statistical analysis.
(B)
t-AMPK

## Slide 7
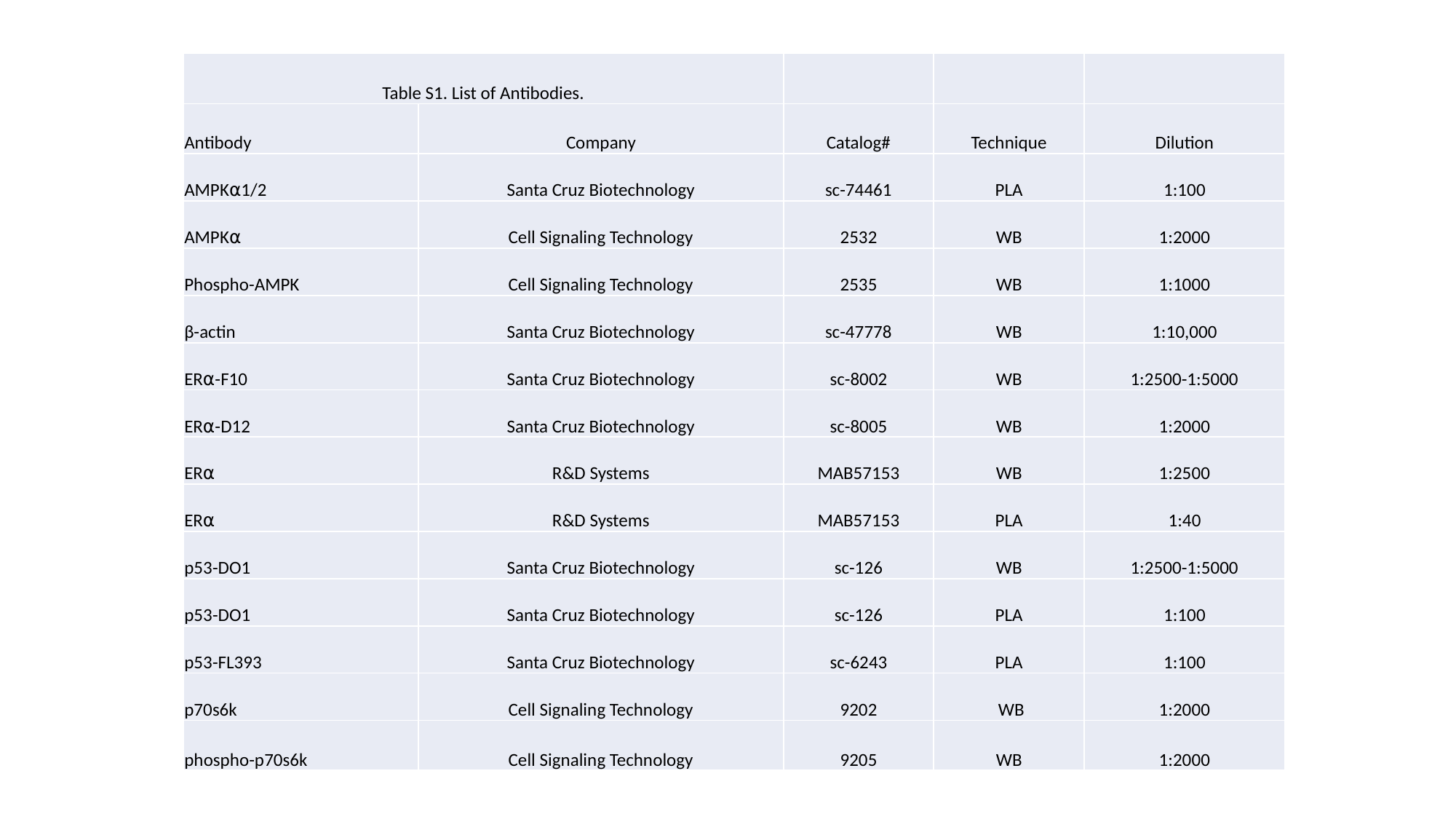

| Table S1. List of Antibodies. | | | | |
| --- | --- | --- | --- | --- |
| Antibody | Company | Catalog# | Technique | Dilution |
| AMPK⍺1/2 | Santa Cruz Biotechnology | sc-74461 | PLA | 1:100 |
| AMPK⍺ | Cell Signaling Technology | 2532 | WB | 1:2000 |
| Phospho-AMPK | Cell Signaling Technology | 2535 | WB | 1:1000 |
| β-actin | Santa Cruz Biotechnology | sc-47778 | WB | 1:10,000 |
| ER⍺-F10 | Santa Cruz Biotechnology | sc-8002 | WB | 1:2500-1:5000 |
| ER⍺-D12 | Santa Cruz Biotechnology | sc-8005 | WB | 1:2000 |
| ER⍺ | R&D Systems | MAB57153 | WB | 1:2500 |
| ER⍺ | R&D Systems | MAB57153 | PLA | 1:40 |
| p53-DO1 | Santa Cruz Biotechnology | sc-126 | WB | 1:2500-1:5000 |
| p53-DO1 | Santa Cruz Biotechnology | sc-126 | PLA | 1:100 |
| p53-FL393 | Santa Cruz Biotechnology | sc-6243 | PLA | 1:100 |
| p70s6k | Cell Signaling Technology | 9202 | WB | 1:2000 |
| phospho-p70s6k | Cell Signaling Technology | 9205 | WB | 1:2000 |
